# Polar localization of the ATPase ClpV-5 occurs independent of type VI secretion system apparatus proteins in *Burkholderia thailandensis*

**DOI:** 10.1186/s13104-019-4141-3

**Published:** 2019-02-28

**Authors:** Jan Lennings, Christian Mayer, Munira Makhlouf, Heike Brötz-Oesterhelt, Sandra Schwarz

**Affiliations:** 1 Interfaculty Institute of Microbiology and Infection Medicine, Department of Medical Microbiology and Hygiene, University of Tübingen, Tübingen, Germany; 2 Interfaculty Institute of Microbiology and Infection Medicine, Department of Microbial Bioactive Compounds, University of Tübingen, Tübingen, Germany

**Keywords:** *Burkholderia thailandensis*, Host cell, Type VI secretion system, ClpV ATPase

## Abstract

**Objective:**

ClpV, the ATPase of the type VI secretion system (T6SS) recycles cytoplasmic T6SS proteins following effector translocation. Fluorescent protein fusions to ClpV showed that it localizes to discrete and dynamic foci. ClpV-1-sfGFP of the bacterial cell targeting T6SS-1 of *Burkholderia thailandensis* exhibits a virtually random localization, whereas ClpV-5-sfGFP of the T6SS-5 targeting host cells is located at one or both poles. The mechanisms underlying the differential localization pattern are not known. Previous analysis of T6SSs, which target bacterial cells revealed that ClpV foci formation is dependent on components of the T6SS. Here, we investigated if the T6SS-5 apparatus confers polar localization of ClpV-5.

**Results:**

ClpV-5-sfGFP foci formation and localization was examined in a *B. thailandensis* mutant harboring a deletion of the entire T6SS-5 gene cluster. We found that ClpV-5-sfGFP localization to discrete foci was not abolished in the absence of the T6SS-5 apparatus. Furthermore, the number of ClpV-5-sfGFP foci displaying a polar localization was not significantly different from that of ClpV-5-sfGFP expressed in the wild type genetic background. These findings suggest the presence of a T6SS-independent localization mechanism for ClpV-5 of the T6SS-5 targeting host cells.

**Electronic supplementary material:**

The online version of this article (10.1186/s13104-019-4141-3) contains supplementary material, which is available to authorized users.

## Introduction

The type VI secretion system (T6SS) is a widespread nanomachine bearing considerable structural and mechanistic similarity to contractile phage tails [1–3]. T6SSs are employed as offensive or defensive tools to kill prokaryotic or eukaryotic target cells or to acquire metal ions to overcome stress conditions, respectively [4–6]. The T6S apparatus is comprised of a cytoplasmic tubule—made of an inner tube enclosed in a contractile sheath—and an envelope spanning membrane complex that are connected by a base plate [7]. Contraction of the sheath acts as a molecular spring that pushes the inner tube tipped with a spike protein into the extracellular milieu or into the target cell, thereby translocating effector proteins [8]. The contracted sheath remains in the cytoplasm and is recycled by the ring-forming AAA^+^ ATPase ClpV enabling a new assembly-contraction-disassembly cycle [9–11]. ClpV of *Vibrio cholerae* interacts directly with the N-terminus of the sheath protein TssC that is exposed in its contracted state [11–14]. Fluorescent protein fusions to ClpV of T6SSs targeting bacterial cells showed that the protein localizes to dynamic foci, which is dependent on components of the T6SS [9, 15, 16]. Localization dynamics of ClpV foci are associated with lysis or blebbing of the neighboring target cell indicating a prior translocation event [9, 17].

*Burkholderia thailandensis* is a soil saprophyte able to switch to an intracellular life style upon contact with phagocytic and non-phagocytic cells [18, 19]. The bacteria encode multiple T6SSs belonging to the canonical T6SS^i^ subtype [20], [21]. The T6SS-5, which is expressed during infection of host cells, induces the formation of multinucleated giant cells (MNGCs) for intercellular spread of the bacteria and the T6SS-1 is involved in interbacterial interactions [18, 22, 23]. This work extends a previous study showing that the ATPase of the T6SS-1 (ClpV-1) and T6SS-5 (ClpV-5) localizes to distinctly different sites inside *B.* *thailandensis* [16]. In contrast to ClpV-5-GFP foci, which were predominantly found at one or both cell poles, ClpV-1-GFP foci localized in a nearly random manner along the cell length, similar to ClpV of other bacterial cell targeting T6SS. In addition, ClpV-1-GFP foci are more dynamic than ClpV-5-sfGFP foci [16]. The mechanism underlying the differential localization pattern is not known. Here, we imaged a ClpV-5-sfGFP fusion protein in *B. thailandensis* harboring a deletion of the entire T6SS-5 gene cluster to investigate if T6SS-5 apparatus proteins direct ClpV-5 to the pole. We found that the polar localization of ClpV-5 is not dependent on the interaction with other T6SS-5 proteins.

## Main text

### Methods

#### Bacterial strains and growth conditions

*Escherichia coli* DH5α and SM10λpir and *B. thailandensis* E264 were routinely grown in Luria–Bertani (LB) medium at 37 °C supplemented with 200 μg/ml trimethoprim and 0.02% rhamnose when necessary.

#### Construction of plasmids and *B. thailandensis* mutants

*Burkholderia thailandensis* ΔT6SS-5 carrying an unmarked deletion of the T6SS-5 gene cluster (BTH_II0855-BTH_II0873) and *B. thailandensis* expressing a chromosomal fusion of *mCherry* to *clpV*-*5* at the native site (*clpV*-*5*-*mCherry*) were generated using the suicide vector pJRC115 as described previously [23, 24]. The mini-Tn*7* transposon delivery plasmid pUC18T-mini-Tn*7*T-Tp was used for expression of *clpV*-*5*-*sfgfp* from a neutral chromosomal site in ΔT6SS-5. The gene *clpV*-*5* (BTH_II0864) fused to *sfgfp* was cloned into pUC18T-mini-Tn*7*T-Tp::*P*_S12_ to express *clpV*-*5*-*sfgfp* under control of the constitutive ribosomal promoter *P*_S12_ (BaseClear) [23, 25]. Transformation of *B.* *thailandensis* ΔT6SS-5 with this and the transposase helper plasmid pTNS3 yielded the mutant ΔT6SS-5 *att*Tn*7*::*P*_S12_-*clpV*-*5*-*sfgfp*, which we termed T6SS-5^−^ *clpV*-*5*-*sfgfp*. *B.* *thailandensis* expressing a chromosomal fusion of *clpV*-*5*-*sfgfp* (T6SS-5^+^ *clpV*-*5*-*sfgp*) was generated in previous work [16]. The genes *virA* (BTH_II0871) and *virG* (BTH_II0872) encoding the two component system VirAG were cloned into the expression vector pSCrhaB2 carrying a rhamnose inducible promoter (p::*virAG*) [26].

#### Infection of RAW 264.7 macrophages and fluorescence microscopy

**Fig. 1:**
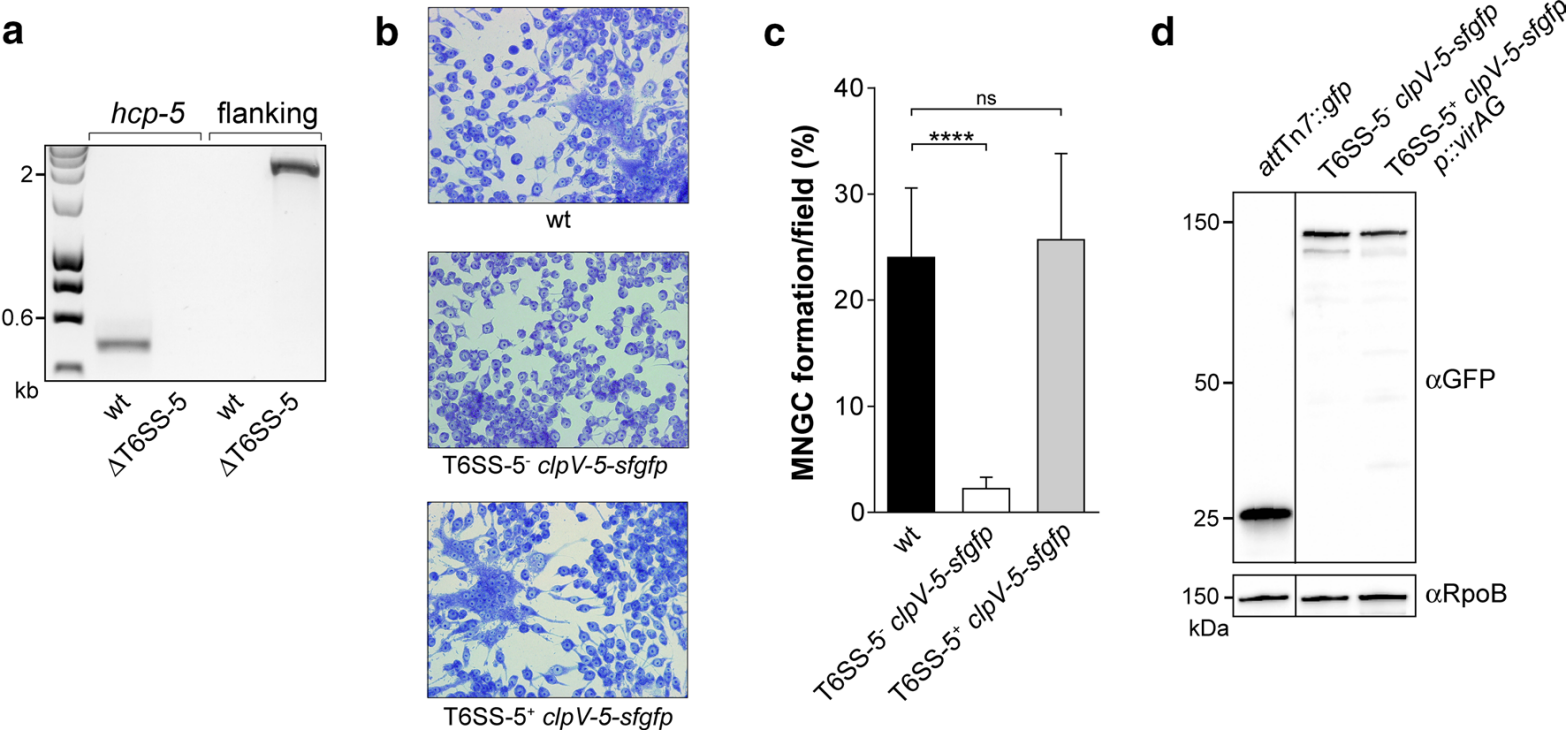
Verification of *B. thailandensis* T6SS-5^−^ *clpV*-*5*-*sfgfp* and T6SS-5^+^ *clpV*-*5*-*sfgfp* mutants. **a** Confirmation of the deletion of the T6SS-5 gene cluster (size: ~ 28 kb) in *B.* *thailandensis* by PCR using genomic DNA of the indicated strains and primers specific for the T6SS-5 gene *hcp*-*5* (yielding a 489 bp product for the wild type and no amplicon for the ΔT6SS-5 mutant) and genes flanking the T6SS-5 gene cluster (yielding a 28,201 bp and 1885 bp product for the wild type and ΔT6SS-5 mutant, respectively). **b** Representative images of RAW264.7 macrophages infected with *B.* *thailandensis* ΔT6SS-5 *att*Tn7::*P*_S12__*clpV*-*5*-*sfgfp* (T6SS-5^−^ *clpV*-*5*-*sfgfp*) and a strain expressing a chromosomal *clpV*-*5*-*sfgfp* fusion (T6SS-5^+^ *clpV*-*5*-*sfgfp*) at MOI 2 for 13 h and stained with Giemsa. **c** Quantification of MNGC formation of RAW264.7 macrophages infected with the indicated *B.* *thailandensis* strains and stained as described in B. The ability of the T6SS-5^+^ *clpV*-*5*-*sfgfp* mutant to induce MNGC formation shows that ClpV-5 is functional when fused to sfGFP. The data shown are mean values +SD based on two experiments performed in triplicate and three randomly selected microscopic fields per sample (N > 7578 nuclei). ns, not significant/*P* = 0.498 (t-test); ****P < 0.0001 (Welch’s t-test). **d** Detection of ClpV-5-sfGFP fusion proteins in whole cell lysates of the indicated *B.* *thailandensis* strains by Western blot analysis using α-GFP antibody (MW: GFP, 27 kDa; ClpV-5, 101 kDa) and as loading control α-RpoB antibody (MW: RpoB, 153 kDa). The bacteria were grown to an OD_600nm_ of 1.0 in LB broth. The genes BTH_II0871 and BTH_II0872 encoding the two component system VirAG were overexpressed (p::*virAG*) in *B.* *thailandensis* T6SS-5^+^ *clpV*-*5*-*sfgfp* to induce production of the fusion protein outside the host cell environment

The RAW 264.7 murine macrophage cell line (ATCC) and the Hela epithelial cell line (ATCC) were maintained in high glucose DMEM supplemented with 1 mM sodium pyruvate and 10% fetal bovine serum (Gibco) at 37 °C and 5% CO_2_. On the day before the experiment 1 × 10^5^ macrophages and 5 × 10^4^ Hela cells were seeded on glass cover slips in 24 well plates. The cells were infected with *B. thailandensis* wild type and mutants harvested from exponential phase cultures at multiplicity of infection (MOI) 2 (macrophages) and 50 (Hela cells) and incubated for 1 h. The medium was replaced with fresh DMEM containing 100 μg/ml imipenem followed by 13 h incubation. At this time point the macrophages were stained with Giemsa (Sigma) and viewed with a Olympus BX51 microscope and a 20 × (for quantification of MNGC formation) and 40 × (for images shown in Fig. 1b) objective. MNGC formation was quantified using the formula: (number of nuclei within MNGCs/total number of nuclei) × 100. For epifluorescence microscopy, macrophages were infected with *B.* *thailandensis* at MOI 50 for 6 h and fixed with 4% formaldehyde. F-actin and DNA was stained with Texas Red-X Phalloidin or Alexa Fluor 488 Phalloidin and 4′,6-diamidino-2-phenylindole (DAPI), respectively. Images of cells were acquired with a Nikon Eclipse Ti-E equipped with a CCD Hamamatsu Orca Flash 4.0 camera and a CFI Plan-Apo DM 100×/1.45 Oil Ph3 objective.

#### Western blot

*Burkholderia thailandensis* strains were grown in LB broth supplemented with 0.02% rhamnose and 200 μg/ml trimethoprim where necessary. A 1 ml aliquot of bacterial cultures grown to an OD_600nm_ of 1.0 was centrifuged and the cell pellet was resuspended in H_2_O and Laemmli sample buffer. Following SDS PAGE the samples were transferred to nitrocellulose membranes, which were blocked with 5% skim milk and probed with the primary antibodies mouse monoclonal anti-GFP (Thermo Fisher; MA5-15256) or mouse monoclonal anti-RNA polymerase beta (Thermo Fisher; MA1-25425). HRP-conjugated rabbit anti-mouse (Thermo Fisher; 31457) was used as secondary antibody. Blots were developed using Clarity Western ECL substrate (Biorad).

#### Statistical analysis

The t-test or Welch’s t-test was performed to test the difference between two means as indicated in the figure legends. A *P* value of ≤ 0.05 was considered statistically significant.

### Results and discussion

To examine whether ClpV-5 localizes to the pole by interacting with protein(s) of the T6SS-5 apparatus, we constructed an unmarked deletion mutant of the entire T6SS-5 gene cluster (BTH_II0855–BTH_II0873) in *B.* *thailandensis*. This mutant lacks all components of the T6SS-5 secretion apparatus. ClpV-5 localization analysis in the absence of T6SS-5 components was performed by integrating a *clpV*-*5*-*sfgfp* fusion gene into the chromosome of the ΔT6SS-5 mutant under control of a constitutive promoter using the mini-Tn*7* system (T6SS-5^−^ *clpV*-*5*-*sfgfp*). Deletion of the T6SS-5 gene cluster was confirmed by PCR and the inability of the T6SS-5^−^ *clpV*-*5*-*sfgfp* mutant to induce MNGC formation of RAW 264.7 macrophages (Fig. 1a–c). As control, bacteria expressing a chromosomal *clpV*-*5*-*sfgfp* fusion in the wild type genetic background were used (T6SS-5^+^ *clpV*-*5*-*sfgfp*). The capability of this mutant to mediate MNGC formation at levels similar to the wild type demonstrates that ClpV-5 is functional when fused to sfGFP (Fig. 1b, c). Production of the ClpV-5-sfGFP fusion protein by *B. thailandensis* T6SS-5^−^ *clpV*-*5*-*sfgfp* and T6SS-5^+^ *clpV*-*5*-*sfgfp* was confirmed by Western blot analysis (Fig. 1d). To this end, cell lysates of bacteria grown in LB broth were used. Transcription of *clpV*-*5*-*sfgfp* in T6SS-5^+^ *clpV*-*5*-*sfgfp* was achieved by overexpression of the two component regulatory genes *virAG* (p::*virAG*) previously shown to stimulate the T6SS-5 under conditions that lack the native host cell derived activation signal [16, 27, 28]. Furthermore, we investigated the localization of ClpV-5 fused to the monomeric fluorescent protein mCherry during infection of host cells. ClpV-5-mCherry localizes to single discrete foci at the pole of *B. thailandensis* thus verifying the localization pattern observed for ClpV-5-sfGFP (Additional file 1: Figure S1).

**Fig. 2:**
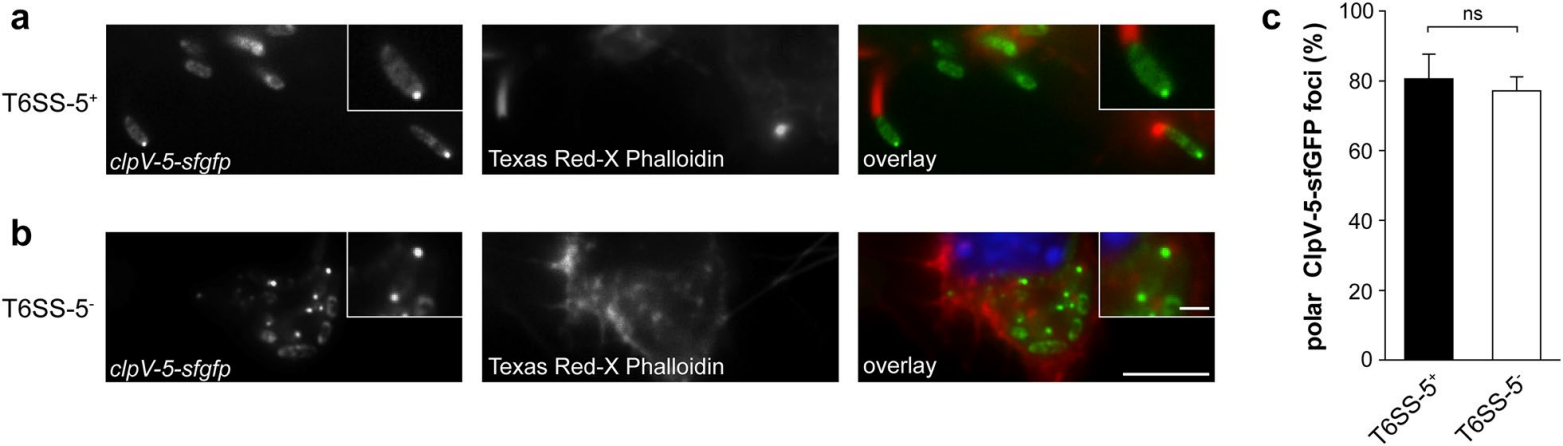
Polar localization of ClpV-5-sfGFP within *B. thailandensis* is not abolished in the absence of all T6SS-5 components. **a**, **b** RAW264.7 macrophages were infected with *B.* *thailandensis* **a** expressing a chromosomal *clpV*-*5*-*sfgfp* fusion in the wild type background (T6SS-5^+^ *clpV*-*5*-*sfgfp*) and **b** carrying a deletion of the entire T6SS-5 gene cluster and expressing *clpV*-*5*-*sfgfp* constitutively from a neutral site on the chromosome (T6SS-5^−^ *clpV*-*5*-*sfgfp*) at MOI 50 for 6 h. Host cell actin and DNA was stained with Texas Red-X Phalloidin and DAPI, respectively. Because the regulatory genes *virA/virG*, which are located within the T6SS-5 gene cluster, are required for activating the actin polymerization gene *bimA*, the T6SS-5^−^ mutant is unable to form actin tails. Scale bars, 5 μm and 1 μm for main figure and insets, respectively. **c** Quantification of ClpV-5-sfGFP foci displaying a polar localization in *B. thailandensis* T6SS-5^+^ *clpV*-*5*-*sfgfp* and T6SS-5^−^ *clpV*-*5*-*sfgfp* during infection of RAW264.7 macrophages. The data shown represent mean values +SD based on two experiments performed in duplicate and three randomly selected microscopic fields per sample (N ≥ 223 foci). ns, not significant/*P* = 0.156 (t-test)

Given that the native environment for T6SS-5 expression and activity is the intracellular milieu of the host cell, we studied the localization of ClpV-5 within *B.* *thailandensis* during infection of RAW264.7 macrophages. The cells were infected with *B.* *thailandensis* T6SS-5^−^ *clpV*-*5*-*sfgfp* and as control with T6SS-5^+^ *clpV*-*5*-*sfgfp*. In the T6SS-5^+^ genetic background giving rise to a complete and functional T6SS-5, the ClpV-5-sfGFP fusion protein displayed the formation of discrete foci and diffuse cytoplasmic localization as reported previously (Fig. 2a) [16]. The majority of ClpV-5-sfGFP foci (81%) exhibited a polar localization (Fig. 2a, c). Interestingly, in the absence of all T6SS-5 components ClpV-5-sfGFP still assembled into foci located at the pole (Fig. 2b). Furthermore, the number of polar ClpV-5-sfGFP foci did not significantly differ between T6SS-5^+^ and T6SS-5^−^ bacteria (Fig. 2c). The non-native chromosomal position of *clpV*-*5*-*sfgfp* in the T6SS-5^−^ mutant did not abolish polar localization of the fusion protein. The data demonstrate that T6SS-5 apparatus protein(s) are not required for polar localization of ClpV-5-sfGFP. Instead, a non-T6SS protein may serve for example as a polar anchor for ClpV-5. The finding that T6SS-5 components are dispensable for ClpV-5 localization is consistent with reports showing that ClpV specifically interacts with the T6 apparatus following a secretion event, i.e. with the contracted sheath [9]. Moreover, ClpV1 of the bacterial cell targeting T6SS of *E.* *coli* does not directly interact with any of the T6SS apparatus components except for TssC [12]. On the other hand, the deletion of components of bacterial cell targeting T6SSs in *V.* *cholerae, Pseudomonas* *aeruginosa* and *Serratia marcescens* abrogated ClpV foci formation and resulted in a diffuse cytoplasmic localization [9, 15, 29–31]. Likewise, the ATPase ClpB of the anti-host but non-canonical T6SS in *Francisella novicida* requires the T6SS protein PdpB for localization [32]. Altogether, the results suggest that complex and distinct localization mechanisms underlie the positioning of the ATPase of bacterial and host cell targeting T6SS.

## Limitations

Fluorescent protein fusion, a widely used method for protein localization studies, was used to determine the subcellular localization of ClpV-5. A limitation of this technique is that the fluorescent protein might alter localization and activity of ClpV-5. The *clpV*-*5*-*sfgfp* fusion was expressed from the native chromosomal *clpV*-*5* locus activated by the native host cell signal and we confirmed that ClpV-5 was functional when fused to sfGFP. Moreover, a previous study utilizing ClpV specific antibodies showed that –like ClpV-5-sfGFP– the native untagged ClpV protein assembles into discrete foci in *V.* *cholerae* [29].

## Additional file

**Additional file 1: Figure S1.** A ClpV-5-mCherry fusion protein localizes to the bacterial cell pole during infection of host cells. Phase contrast and fluorescence microscopy images of Hela cells infected with *B.* *thailandensis* expressing a chromosomal *clpV*-*5*-*mCherry* fusion at MOI 50 for 13 h. Host cell actin was stained with Alexa Fluor 488 Phalloidin. Scale bar, 2 μm.

## Abbreviations

*att*Tn*7*: attachment site of Tn*7*
HRP: horseradish peroxidase
MNGC: multinucleated giant cell
MW: molecular weight
RpoB: RNA polymerase subunit beta
sfGFP: superfolder GFP
