# Supplementary figures and images for: Polar localization of the ATPase ClpV-5 occurs independent of type VI secretion system apparatus proteins in Burkholderia thailandensis
Source: BMC Res Notes. 2019 Feb 28;12:109. doi: 10.1186/s13104-019-4141-3 (PMC6394029; doi:10.1186/s13104-019-4141-3)

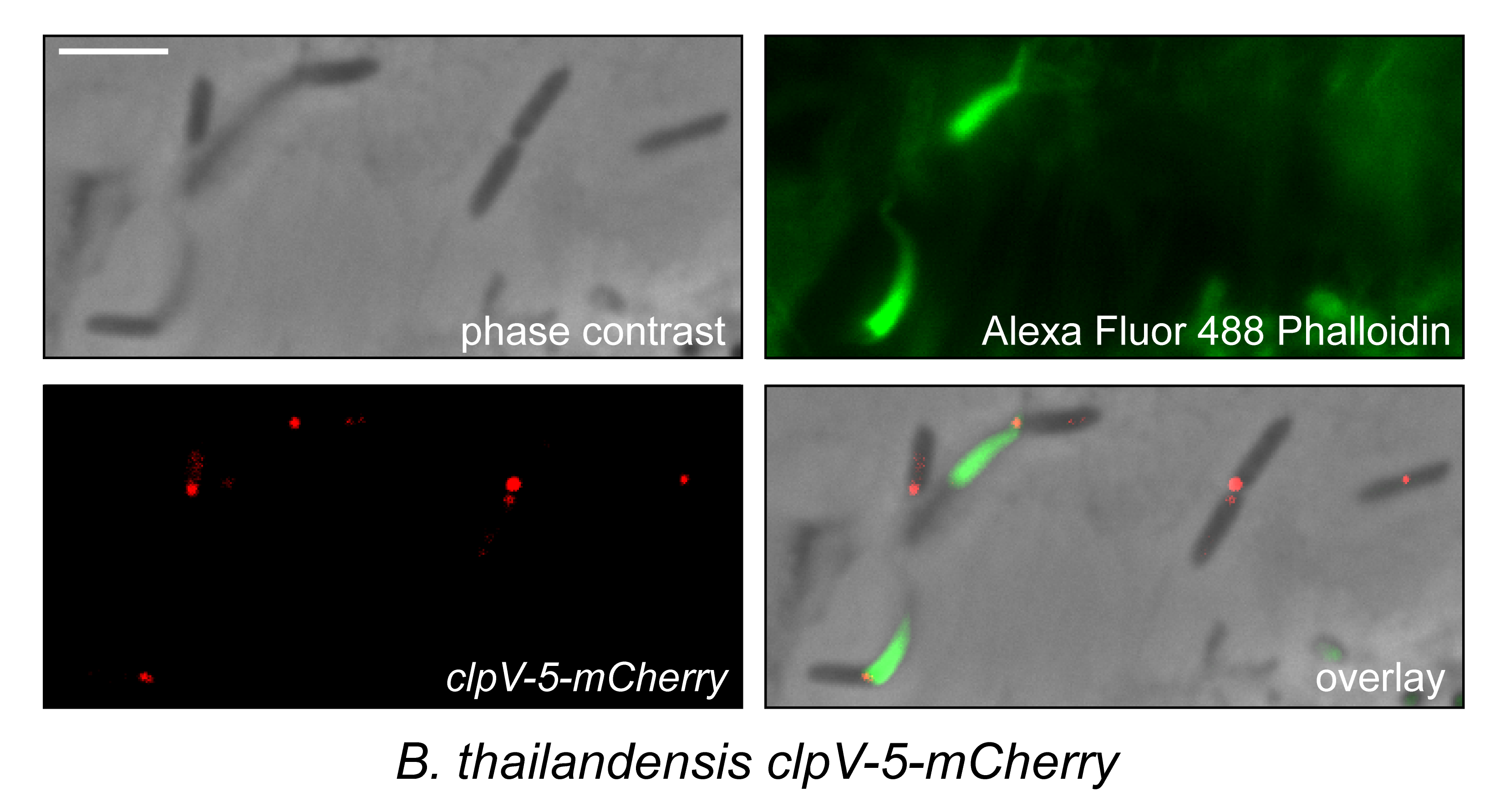

Supplement: Supplementary file 1 — Additional file 1: Figure S1. A ClpV-5-mCherry fusion protein localizes to the bacterial cell pole during infection of host cells. Phase contrast and fluorescence microscopy images of Hela cells infected with B. thailandensis expressing a chromosomal clpV-5-mCherry fusion at MOI 50 for 13 h. Host cell actin was stained with Alexa Fluor 488 Phalloidin. Scale bar, 2 μm. [file 13104_2019_4141_MOESM1_ESM.tif]
